# P100 - Artificial food additives as the cause of chronic soiling in a 14 year old male

**DOI:** 10.1186/2045-7022-4-S1-P155

**Published:** 2014-02-28

**Authors:** Chrys Tsakona

**Affiliations:** 1 Dudley Group of Hospitals NHS Trust, Dudley, United Kingdom

## 

A 14 year old boy was referred by his GP after persisting requests by his mother for investigation of a food allergy as a possible cause for 6-year history of soiling. He was not conscious of his bowels being opened and this would happen several times a day. Paediatricians claimed a psychological reason, which the patient and his mother denied categorically.

• Symptoms inconsistent with allergy

• The Neurologists excluded *spina bifida*

• Returned to the Immunology clinic and it was revealed that he had been referred to the Paediatricians 7 years ago for bloating, flatulence and IBS symptoms and had been diagnosed as having constipation and treated for it ever since. His mother insisted that the anti-constipation treatment aggravated the flatulence and bloating, but the treatment was still recommended as essential.

• On direct questioning his diet it appeared that his diet, although controlled in terms of calories and fat content (he was overweight for his age), was rich in food chemicals and especially diet drinks, so a strict additive-free diet was advised for 6 months (most food chemicals take 2 months to be excreted from the body).

His symptoms resolved completely within a few weeks.

In experimental animals artificial food additives are known to influence the lower gastrointestinal tract under some defined conditions, resulting in morphological alterations in the mucosa and other tissues, deranged absorption and excretion of nutrients, and, in some cases, injury to other organs and tissues as a secondary phenomenon. This influence has been definitely reported in humans only for sulphites which can cause loose stool and diarrhoea. These chemicals are contained in alcohol and also cause the “*salad bar syndrome*”.

In this case the diet did not obviously contain sulphites, suggesting that the complete remission of symptoms was achieved by the exclusion of the common food chemicals usually contained in a teenager’s diet.

